# Forensic Application of Epidermal Ubiquitin Expression to Determination of Wound Vitality in Human Compressed Neck Skin

**DOI:** 10.3389/fmed.2022.867365

**Published:** 2022-04-13

**Authors:** Siying Zhang, Yuko Ishida, Akiko Ishigami, Mizuho Nosaka, Yumi Kuninaka, Satoshi Hata, Hiroki Yamamoto, Yumiko Hashizume, Jumpei Matsuki, Haruki Yasuda, Akihiko Kimura, Fukumi Furukawa, Toshikazu Kondo

**Affiliations:** Department of Forensic Medicine, Wakayama Medical University, Wakayama, Japan

**Keywords:** ubiquitin, compression, neck skin, immunohistochemistry, forensic pathology

## Abstract

Ubiquitin is a member of the heat shock protein family and is rapidly induced by various types of stimuli, including ischemic and mechanical stress. However, its significance in determining wound vitality of neck compression skin in forensic pathology remains unclear. We immunohistochemically examined the expression of ubiquitin in the neck skin samples to understand its forensic applicability in determining wound vitality. Skin samples were obtained from 53 cases of neck compression (hanging, 42 cases; strangulation, 11 cases) during forensic autopsies. Intact skin from the same individual was used as the control. Ubiquitin expression was detected in 73.9% of keratinocytes in intact skin samples, but only in 21.2% of keratinocytes in the compression regions, with statistical differences between the control and compression groups. This depletion in the case of neck compression may be caused by the impaired conversion of conjugated to free ubiquitin and failure of *de novo* ubiquitin synthesis. From a forensic pathological perspective, immunohistochemical examination of ubiquitin expression in the skin of the neck can be regarded as a valuable marker for diagnosing traces of antemortem compression.

## Introduction

In forensic practices, differentiating antemortem injury from postmortem damage is one of the important issues, which is a classical but still modern topic (1–3). Thus, there are numerous forensic studies exploring available markers for the determination of wound vitality using biochemical, histochemical, immunohistochemical and molecular biological techniques (4–6). Moreover, there are several recent studies focusing on omics sciences and micro RNA (7, 8).

Fatal asphyxia due to neck compression is often encountered during forensic autopsies. Neck ligature marks can result from various lesions, including manual strangulation, ligature strangulation, strangulation, direct striking, armlocks, and cord entanglement (9, 10). Ligature mark is the most important finding on the neck for forensic pathologists. The mark is in the form of a furrow or groove in the tissue, pale in color and may later change from tan to dark brown. The dryness and desiccation of the abraded skin makes the mark hard and parchment-like (11). In contrast, if a soft material, such as a towel, is used, or if a beard or a part of the cloth is between the ligature and the skin, the ligature mark may be faint or inconspicuous. Microscopy of the thyroid and salivary glands usually reveals focal interstitial hemorrhages, and the lymph gland shows congestion, supporting the antemortem nature of neck compression (12). However, only few studies have focused on the relationship between the ligature compression and neck tissue, and very few markers have been investigated as an evidence for neck compression (13–15).

Ubiquitin is an 8.5 kDa protein containing 76 amino acids and is commonly found in eukaryotic cells (16). Ubiquitin has seven lysine and methionine sites at its N-terminus, which tend to get self-ubiquitinated and extend to form different types of polyubiquitin chains (17). Ubiquitination is closely associated with many cellular processes such as cell cycle regulation, immune response, inflammatory response, and apoptosis. Major chronic degenerative diseases in humans, including Alzheimer’s disease, Parkinson’s disease, and amyotrophic lateral sclerosis, are primarily associated with the nervous system, and ubiquitin is incorporated into many inclusion bodies that characterize these neurodegenerative diseases (18). Additionally, ubiquitin is involved in the response to acute cell injury. Transcription of ubiquitin mRNA in mammalian cells is induced by heat shock and other stresses (19, 20). In contrast, short-term ischemia causes depletion of free ubiquitin in gerbil hippocampal neurons, which are the most vulnerable to ischemic injury (21). Moreover, ubiquitin gene expression after ischemia/reperfusion has been studied in the rat brains, where it was observed to initially decrease after reperfusion; however, the expression increased after the blood flow was restored (22), suggesting that ubiquitin expression may serve as an indicator of the ischemic stress.

In the field of forensic pathology, there are several studies have investigated whether ubiquitin expression can be used as an indicator for forensic diagnosis. An immunohistochemical study of ubiquitin in the human locus coeruleus has shown that the number of neurons with ubiquitin expression is significantly higher in the case of long-term death struggles (23). In human kidney tissues, ubiquitin-immunopositive tubular epithelial cells are higher than the other groups involving subjects who died due to fire, blunt injury, sharp injury, and fatal hypothermia, suggesting that ubiquitin positivity is a characteristic of death due to injury and hypothermia (24, 25). In addition, the ubiquitin immune responsiveness of pigmented substantia nigra neurons in the midbrain has been suggested to be triggered by severe, deadly stress from asphyxia, drowning, and fire (26, 27). We also have previously reported that ubiquitin would be one of the markers for wound age estimation (28). However, there have been no immunohistochemical or forensic diagnostic studies pertaining to ubiquitin using compressed neck skin. In this study, we investigated the immunohistochemical expression of ubiquitin in neck skin specimens from autopsy cases and discussed whether it can be a useful marker for the forensic diagnosis of compression.

## Materials and Methods

### Antibodies

The following polyclonal antibodies (pAbs) were used for immunohistochemical analysis in the present study: rabbit anti-ubiquitin pAbs (UBA52 Ab, AF0289, Affinity Biosciences, Jiangsu, China).

### Human Ligature Marks

A total of 53 ligature marks (hanging, 42 cases; strangulation, 11 cases) with a postmortem interval of <96 h were obtained from forensic autopsies at our institute. In each case, the cause of death was carefully determined based on autopsy, histopathological findings and toxicological data. Intact skin from the same individual was used as the control. The detailed profiles of all cases (sex, age, and postmortem intervals) are shown in Table 1.

**TABLE 1:** Cases profile.

Number	Male/Female	Age (y)		Postmortem interval (h)	
		Range	Mean	Range	Mean
53	32/21	16–90	59.2	10–84	35.8

### Immunohistochemical Analysis

Skin specimens were fixed in 4% formaldehyde solution buffered with PBS for 4–7 days, embedded in paraffin, and sectioned at a thickness of 4 μm. Briefly, deparaffinized sections were incubated with PBS containing 1% normal goat serum and 1% bovine serum albumin (BSA) to reduce non-specific reactions. Thereafter, the sections were further incubated with anti-ubiquitin pAbs (dilution 1:100) for 12–17 h at 4°C. After incubation with biotinylated secondary antibodies, immune complexes were visualized using Catalyzed Signal Amplification System (Dako, Kyoto, Japan) according to the manufacturer’s instructions. As a negative control, sections were incubated with normal rabbit serum instead of the primary antibodies; and no positive signal was detected in this case, thus indicating the specificity of the antibodies.

### Morphometrical Analysis

To evaluate ubiquitin expression in the skin, the ratio of ubiquitin-positive keratinocytes to the total number of corresponding keratinocytes wase calculated in five randomly selected high-power fields (×400). The average values were evaluated as an indicator for ubiquitin expression. Morphometric evaluation was blindly performed by two investigators without the prior knowledge of the samples.

### Statistical Analysis

The mean and standard error of the means (SEM) were calculated. Statistical analysis was performed using analysis of variance or Mann-Whitney *U*-test. Statistical significance was set at *P* < 0.05. Correlation analysis was performed using the non-parametric Spearman’s correlation coefficient. Statistical significance was set at *P* < 0.05.

### Ethical Approval

This study was approved by the Research Ethics Committee of Wakayama Medical University (No. 3313). All the procedures were performed in accordance with the Declaration of Helsinki Principles. Moreover, this study was conducted using autopsy records from the past, and we could not obtain informed consent from the bereaved family for the use of these records. Therefore, in accordance with the “Ethical Guidelines for Medical Research Involving Human Subjects (enacted by the Ministry of Health, Labor, and Welfare in Japan), section 12–1 (2) (a) (c).” Since this was a de-identified retrospective study of archived autopsy-derived tissue, the review board of the Research Ethics Committee of Wakayama Medical University waived the need for written informed consent from the relatives of the individuals studied.

## Results

### Immunohistochemical Analysis of Ubiquitin in Autopsy Samples

**FIGURE 1:**
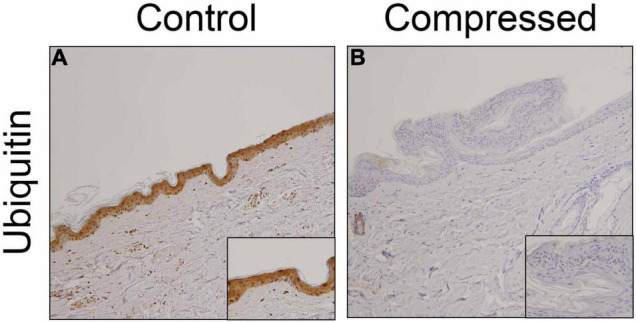
Immunohistochemical analysis. Immunohistochemical analysis were performed by using anti-ubiquitin pAb in the human skin samples. **(A)** control; **(B)** compressed neck skin. Original magnification, × 200; inset, × 400.

We examined the distribution of ubiquitin in the skin samples. Consistent with previous observations (28), ubiquitin-positive signals were observed predominantly in predominantly keratinocytes of uninjured skin samples (Figure 1A). However, in most ligature marks, ubiquitin was not detected in the keratinocytes (Figure 1B).

### The Expression of Ubiquitin Was Lower in Neck Compression Cases

**FIGURE 2:**
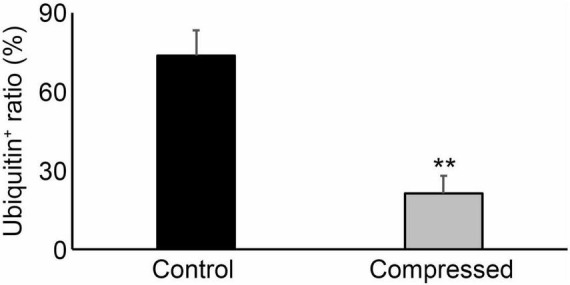
The ratio of ubiquitin positives in the corresponding keratinocytes in the skin sample. **P < 0.01.

**FIGURE 3:**
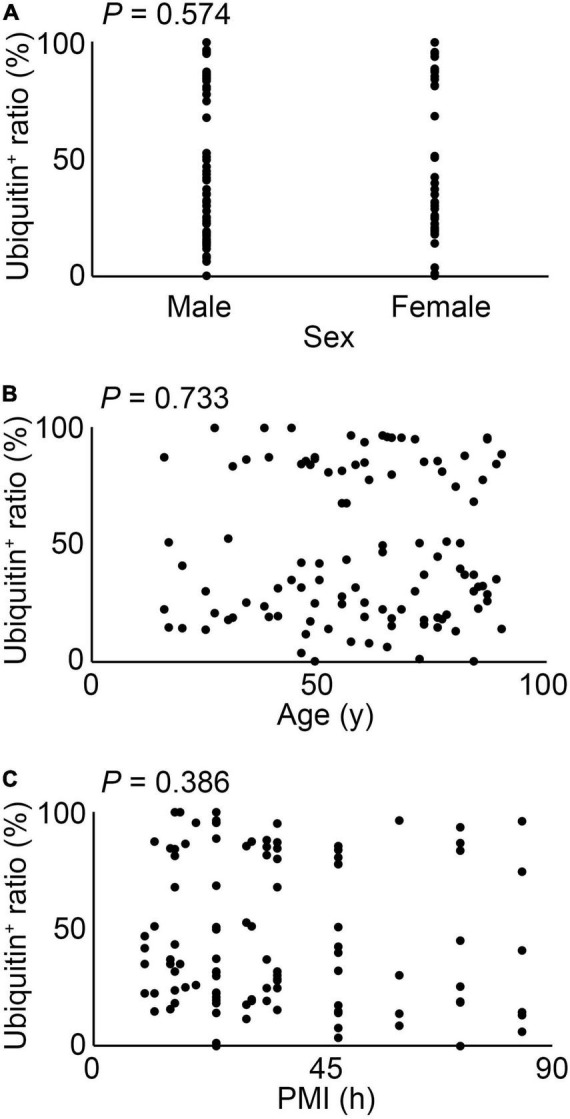
The relation between sex **(A)**, age **(B)** or postmortem intervals (PMI) **(C)** and ubiquitin expression in all cases. These results were obtained with Spearman’s correlation coefficient by rank test.

As shown in Figure 2, the ratio of ubiquitin expression in keratinocytes was significantly suppressed in compressed skin samples, compared to that in the control samples. There were no significant differences among sex, age, and postmortem intervals in terms of ubiquitin expressions (Figure 3).

## Discussion

It is an essential work to determine wound vitality and wound age in forensic autopsy cases. To achieve the purpose, advanced biological techniques are applied to forensic pathology (4–8). In forensic medicine, the compression mark on the neck is one of the most important criteria for determining whether the neck has been affected by the contractile force. Asphyxiation in the forensic context is associated with mechanical asphyxia through various mechanisms such as strangulation, hanging, smothering, choking and aspiration. Direct causes of death include systemic hypoxia due to airway obstruction, as well as possible neurological effects of cerebral ischemia and neck pressure. In general, it is not difficult to observe such marks upon gross examination. However, in certain situations, such as when the force is weak or the vital reaction is uncertain, it may be necessary to confirm the presence of the compression mark using a staining method during the histological examination of the neck skin. Immunohistochemical analyses may provide reliable information for the estimation of vitality using compression marks (2, 3, 15).

There are lot of studies on the determination of wound vitality of ligature marks (15). Turillazzi et al. have investigated the immunohistochemical expression of various cytokines in skin specimens from autopsy cases of death by hanging (29). Previous studies have reported that high expression of IL-15 in the skin of the neck may be a reliable marker of ligature mark vitality. IL-15 is known to activate neutrophils, which are major players in inflammation of damaged tissues. It has been reported that neutrophils were first observed in human skin wounds aged about 20–30 min (30). On the other hand, Kondo et al. have demonstrated that neutrophils were observed primarily at wound sites aged approximately 4–12 h, and were a source of cytokines and chemokines such as IL-1α, IL-8, CCL2, and CCL3 (31, 32). In an experimental study of cytokine expression during skin wound healing in mice, infiltration of numerous neutrophils producing IL-1α, IL-1β, IL-6, and TNF-α was observed at the wound sites 3 and 6 h after injury (33). Therefore, it has been suggested that inflammatory cells and cytokines may act as markers for the determination of wound age and vitality (34–36). Actually, several lines of accumulating evidence implied that IL-1β, one of the representative inflammatory cytokines, would be a candidate molecule for the discrimination between antemortem-postmortem hangings in both animal experiments and human samples. Grellner could confirm that IL-1β immunostaining of epidermal cells was a useful tool to discriminate antemortem and post-mortem hanging (37, 38).

Additionally, Maiese et al. demonstrated that intracytoplasmic depletion of FLIP playing as an inhibitor of apoptosis was evident in the epidermal layers of antemortem neck compression (39). Pérez et al. (40) reported a higher frequency of cells positive to cathepsin D and P-selectin was found in subcutaneous injured skin. Tryptase and CD15 were also useful for the determination of wound vitality in ligature marks (29). Focardi et al. (41, 42) focused on Langerhans cells determined by both antigens of MHC-II and CD1A and revealed that Langerhans cells and their-derived iNOS was significantly higher in ligature marks with vitality. Alternatively, De Matteis et al. (43) examined neck muscle tissues not neck skin samples, and evaluated the use of Troponin I—fast skeletal muscle (TNNI2) to perform differential diagnoses about vitality in suicide by hanging and simulated hanging.

Prangenberg et al. (4) showed heat shock protein was widely applied to forensic pathology such as fire-related death, hypothermia, cardiac death, drowning, excited delirium, trauma and SIDS. Ubiquitin is known as a heat-shock protein induced by several stress conditions (18). Therefore, ubiquitin may contribute to the degradation of denatured proteins produced under various stress conditions. Several lines of accumulating evidence demonstrated the availability of ubiquitin in the postmortem forensic diagnosis of pathophysiology such as asphyxia, drowning, fire, and hypothermia (24–27). Moreover, only our group examined the expression of ubiquitin in human mechanical skin wounds such as stab wounds, cut wounds, surgical wounds and lacerations (28). Subsequently, we have observed an increase of ubiquitin expression in antemortem skin wounds, and demonstrated that a significant ubiquitin positivity rate of above 30% in human skin wounds may indicate the wound age to be of 7–14 days (28). These observations prompt us to examine the expression of ubiquitin in compressed neck skin samples with a hypothesis that ubiquitin expression might be enhanced after antemortem compression. Unexpectedly, we have found the suppression of ubiquitin expression in compressed neck skin samples, compared with intact skin samples. Actually, in the present study, we found that 73.9% of the keratinocytes in the control specimens were ubiquitin-positive, whereas only 21.2% of the keratinocytes in the compressed specimens were ubiquitin-positive with a statistical difference. These observations were similar to those of Maiese’s study that intracytoplasmic depletion of FLIP was found in the epidermal layers of antemortem ligature marks (39).

Ubiquitin gene expression after ischemia/reperfusion has been studied in the rat brains, wherein it was observed to initially decrease after reperfusion, but increased before the blood flow was restored to normal levels (22). These results suggest that ubiquitin may act as a useful marker of ischemic stress. In addition, Morimoto et al. have demonstrated that short-term ischemia causes depletion of free ubiquitin in gerbil hippocampal neurons, which are the most vulnerable to ischemic injury (21). Thus, the discrepancy would result from the difference of wound type between previous (28) and present studies. Although our previous study examined open skin wounds such as cutting and stabbing (28), we employed compressed skin samples indicating closed skin wounds in the present study. Compression to the neck skin can cause more severe local ischemia, eventually resulting in the decrease of ubiquitin expression at the compressed skin area including ligature marks. In other words, a substantial reduction in ubiquitin expression in the skin of the neck may be characteristic of the compression.

From a forensic safety standpoint, it is not sufficient to make a diagnosis using a single marker. For example, immunohistochemical detection of aquaporin-3 (AQP3) in the skin of the neck can be considered a valuable marker for diagnosing traces of antemortem compression (14). Thus, it is emphasized that several different markers reported previously should be examined in forensic practices in order to prevent over-diagnosis or missing of wound vitality. In addition to examining the skin tissue of the neck, markers of neck compression of lung tissue were examined in another study. There were partial differences in the level of immunohistochemical staining for AQP5 among the causes of death such as choking, choking, and sudden cardiac death (44). Moreover, increased thyroglobulin, total T3, and free T3 levels in postmortem blood samples may suggest neck compression (45–47). Recently, Neri et al. showed an increase in the expression of miRNAs recognized as regulators of the inflammatory response in skin lesions such as miR125a-5p and miR125b-5p, implying that regulation of miRNAs as new tool for cutaneous vitality lesions demonstration in ligature marks (48).

## Conclusion

We have showed that the detection of ubiquitin in the neck skin is possible with the accuracy required for forensic purposes. This fact is especially true for soft-marks, which are particularly difficult to assess based on gross examination and conventional histological analysis using HE staining.

## Data Availability Statement

The raw data supporting the conclusions of this article will be made available by the authors, without undue reservation.

## Ethics Statement

The studies involving human participants were reviewed and approved by Research Ethics Committee of Wakayama Medical University (No. 3313). Written informed consent from the participants’ legal guardian/next of kin was not required to participate in this study in accordance with the national legislation and the institutional requirements.

## Author Contributions

SZ, YI, and TK formulated the hypothesis and designed the project. SZ, MN, YK, and AK performed the main experiments. AI, SH, and HiY provided technical assistance and discussion. YH, JM, and HaY helped with some experimental procedures. YI and TK oversaw the experiments and provided the main funding for the project. YI, FF, and TK participated in writing the manuscript. All authors contributed to the article and approved the submitted version.

## Conflict of Interest

The authors declare that the research was conducted in the absence of any commercial or financial relationships that could be construed as a potential conflict of interest.

## Publisher’s Note

All claims expressed in this article are solely those of the authors and do not necessarily represent those of their affiliated organizations, or those of the publisher, the editors and the reviewers. Any product that may be evaluated in this article, or claim that may be made by its manufacturer, is not guaranteed or endorsed by the publisher.
